# Association Among Blood Transfusion, Postoperative Infectious Complications, and Cancer-Specific Survival in Patients with Stage II/III Gastric Cancer After Radical Gastrectomy: Emphasizing Benefit from Adjuvant Chemotherapy

**DOI:** 10.1245/s10434-020-09102-4

**Published:** 2020-09-14

**Authors:** Hua Xiao, Yanping Xiao, Pan Chen, Hu Quan, Jia Luo, Gang Huang

**Affiliations:** 1 Department of Hepatobiliary and Intestinal Surgery, Hunan Cancer Hospital and the Affiliated Cancer Hospital of Xiangya School of Medicine, Central South University, Changsha, Hunan China; 2 Department of Gastroduodenal and Pancreatic Surgery, Hunan Cancer Hospital and the Affiliated Cancer Hospital of Xiangya School of Medicine, Central South University, Changsha, Hunan China; 3 Department of Admissions and Employment, Changsha Health Vocational College, Changsha, Hunan China; 4 Department of Orthopedics, Hunan Cancer Hospital and the Affiliated Cancer Hospital of Xiangya School of Medicine, Central South University, Changsha, Hunan China

## Abstract

**Objectives:**

This study was designed to investigate the potential additive influence of perioperative blood transfusion (BTF) and postoperative infections on cancer-specific survival (CSS) in patients with stage II/III gastric cancer (GC) after radical gastrectomy.

**Methods:**

The medical records of 2114 consecutive stage II/III GC patients who underwent curative resection and planned to receive adjuvant chemotherapy (AC) were retrospectively reviewed. The independent predictive factors for infections were identified using univariate and multivariate analyses. Cox regression analysis was used to assess any associations between BTF, infection and CSS.

**Results:**

A total of 507 (24.0%) received perioperative BTF and 148 (7.0%) developed infections with BTF being identified as an independent predictor for infections. Both BTF and infections independently predicted poor CSS (hazard ratio [HR]: 1.193, 95% confidence interval [CI] 1.007–1.414; HR 1.323, 95% CI 1.013–1.727) and an additive effect was confirmed as patients who had both BTF and infection had even worse CSS. Further stratified analyses showed that complete AC (≥ 6 cycles) could significantly improve CSS in patients who had BTF and/or infection, which was comparable to those without BTF and/or infection (*P* = 0.496).

**Conclusions:**

Infection was the most common complication after gastrectomy and BTF was identified as an independent risk factor. BTF was associated with shorter CSS in stages II/III GC, independent of infections, and receiving BTF and developing infections had an additive effect that was associated with even worse CSS. However, complete AC could significantly improve CSS in these patients. Thus, strategies designed to ensure the completion of AC, such as neoadjuvant chemotherapy, should be further investigated.

**Electronic supplementary material:**

The online version of this article (10.1245/s10434-020-09102-4) contains supplementary material, which is available to authorized users.

Gastric cancer (GC) has been ranked as the fifth most frequent malignancy worldwide and the second leading cause of cancer-related deaths in China.^1^^,^^2^ Surgery is currently the only available curative treatment. Unfortunately, patients with GC in China and western countries are commonly diagnosed at locally advanced stage. In these patients, besides a relatively high incidence of anemia when admitted to hospital, radical gastrectomy with D2 lymphadenectomy sometimes lead to massive hemorrhage even in high-volume centers.^3^^,^^4^ As a result, approximately 20% of GC patients required blood transfusions (BTF) perioperatively.^5^^,^^6^ While BTF is inevitable sometimes, transfusion-related immune modulation (TRIM) and systemic inflammation induced by a BTF not only leads to a higher incidence of postoperative complications, especially infections, but also poorer prognosis.^5^^–^10 In addition, a growing body of evidence supports that postoperative complications adversely affect the long-term survivals of GC patients.^11^^,^^12^ Given that both a BTF and postoperative infectious complications can cause a strong inflammatory response, and results in a pro-tumor environment, we hypothesized that a synergistic unfavorable effect may be observed for survival of GC patients who had both a BTF and infection, a question to the best of our knowledge that has not been previously addressed.

On the other hand, perioperative chemotherapy (including both preoperatively and postoperatively) has been recommended as a standard treatment for advanced GC to improve prognosis.^13^ However, it is not uncommon to encounter patients who are unable to complete the planned adjuvant chemotherapy (AC) due to a poor physical condition or later recovery as a result of postoperative morbidity.^12^^,^^14^ Given that infection was a common and sometimes severe complication following radical gastrectomy for GC, whether it could adversely impact the completion of AC and finally, lead to poorer prognosis, deserves further investigation.

In this retrospective study, we investigated the influence of a BTF on postoperative infections and the potential additive detrimental effect of a BTF and infections on cancer-specific survival (CSS) of patients with stage II/III GC after radical gastrectomy, by analyzing the data from a high-volume center in China. Additionally, we investigated whether completion of AC could compensate for the adverse influence of a BTF and infections on prognosis.

## Methods

### Design and Patients

The medical records of all GC patients undergoing gastrectomy in Hunan Cancer Hospital from November 2010 to May 2019 were retrospectively reviewed. A total of 2306 adult patients (≥ 18 years old) with pathologically diagnosed stage II/III gastric adenocarcinoma experiencing radical gastrectomy (R0 resection and D2 lymph node dissection) were eligible for enrollment in our study. Patients with remnants or recurrence of gastric cancer (*n* = 33), other synchronous malignancies (*n* = 32), and/or missing essential clinical or pathological data (*n* = 127) were excluded, resulting in a total of 2114 patients being enrolled. The study was approved by the ethics committee of the Institutional Review Board of Hunan Cancer Hospital (No. 05 quick review of scientific research in 2020), and written, informed consent for surgery and use of clinicopathological data was obtained from every patient before their operation.

### Perioperative Management and Follow-Up

Experienced surgeons performed or supervised all surgeries. Lymph node dissection and digestive tract reconstruction were performed in keeping with the Japanese gastric cancer treatment guidelines, and classified according to the 8th edition of the American Joint Committee on Cancer TNM staging system.^13^^,^^15^ Some patients with cT3-4/N+ diseases received 2 to 4 cycles of neoadjuvant chemotherapy (NAC) before surgery.^13^ For these who received NAC, pretreatment clinical TNM stage was applied. Patients with locally advanced GC usually required an open procedure, but a small number underwent minimally invasive surgery, including laparoscopy-assisted or total laparoscopic gastrectomy. All patients received a prophylactic antibiotic (generally a second- or third-generation cephalosporin) 30 to 60 min before surgery, which usually lasted for 3 to 5 days after surgery.

Postoperative morbidity and mortality was identified and graded according to the Clavien-Dindo classification system, within 30 days following resection.^16^ Only grade II or greater complications were included for analysis in this study, given the paucity of clinical relevance of grade I complications. AC was usually started about 1 month after gastrectomy using capecitabine/S-1 and oxaliplatin based regimens for about 6 months.^17^^,^^18^ Some patients with stage II disease, or with stage III disease but a relatively poor condition, might receive oral S-1 treatment alone.^13^

Every patient was followed-up at 1 month following resection, every 3 months in the initial 2 years, at half-yearly intervals between years 3 and 5, and yearly thereafter. Physical and laboratory examinations were performed at each follow-up. A computed tomography (CT) scan and/or ultrasonography was recommended at 6-monthly intervals in the first 5 years and endoscopy was performed every 2 years. The latest follow-up date was December 2019.

### Evaluation

The demographic, operative, pathological, and follow-up data were collected from medical records and retrospectively analyzed. As reported in our previous study, perioperative BTF was defined as transfusing packed erythrocytes from admission to discharge for surgery (usually 5 days before surgery and within 14 days thereafter).^6^ Generally, patients were given a BTF if their hemoglobin concentration was < 80 g/L, whereas a BTF also might be given to patients with inappropriate oxygenation or hemodynamic instability, when the hemoglobin concentration range was 80 to 100 g/L, as discussed in our previous study.^6^ The diagnosis of postoperative infectious complications (intra-abdominal infection, pneumonia, etc.) was confirmed in accordance with the Centers for Disease Control and Prevention, and was described in our previous study.^6^^,^^19^

Gastric cancer-specific survival (CSS) was calculated from the time of surgery to death because of GC or the last follow-up. Patients who received ≥ 6 courses of chemotherapy regimens within half a year from the initial AC were considered to have completed AC, given that patients who received < 6 courses of AC had significant poorer outcomes.^17^^,^^20^ To clarify the potential impact of a BTF and/or infection on survival, patients were further classified into four subgroups according to whether they required BTF or experienced infections or not. Patients who required both a BTF and experienced infections [(+)transfusion/(+)infection], required a BTF but did not get an infection [(+)transfusion/(−)infection], had an infection but did not require a BTF [(−)transfusion/(+)infection], and lastly, those patients who did not need a BTF or did not get an infection [(−)transfusion/(−)infection]. To confirm whether a synergistic influence of both transfusion and infection existed for survival, further analysis was performed using the (+)transfusion/(−)infection group as the reference group.^20^^,^^21^

### Statistical Analysis

Data are described as the mean ± SD or numbers (%), and the significance of difference was determined using a Student’s *t* test (or a Mann–Whitney *U* test) or a Chi squared test with Fisher’s exact test, when appropriate. Predictive factors for infections were clarified using multivariate logistic regression analyses. Survival data were analyzed by a Kaplan–Meier curve, and the log-rank test was used to compare subgroups. Predictors that may have influenced CSS were determined by multivariate Cox proportional hazard regression analyses. Multivariate regression analyses using a forward conditional method were performed for factors with a *P* value < 0.05 after univariate analysis. All statistical analysis was conducted using SPSS software (ver. 24.0, IBM Corporation, New York) and a two-sided *P* value < 0.05 was deemed to be statistically significant.

## Results

### Characteristics of Patients

As shown in Table 1,of the 2114 patients included in this study, 1413 (66.8%) were male and 701 (33.2%) female. The mean age was 55.88 years (range, 19–86), and the mean body mass index (BMI) 21.70 kg/m^2^ (range 13.84–35.00). The majority of patients underwent open surgery (74.9%), for a subtotal gastrectomy (71.7%) and patients with stage III disease (73.3%). The mean duration of general anesthesia was 202 min, and the mean estimated intra-operative blood loss was 208 mL. A total of 224 patients (10.6%) received NAC, 507 patients (24.0%) received a perioperative BTF with a median amount of transfused erythrocytes of 4 U (range 1.5–42.5).

**Table 1:** Clinicopathological characteristics of the entire cohort (*n* = 2114)

Variables	*n* (%)
Age; mean (range)	55.88 (19–86)
Body mass index (kg/m^2^); mean (range)	21.70 (13.84–35.00)
Sex	
Male	1413 (66.8%)
Female	701 (33.2%)
American Society of Anesthesiology score	
1	329 (15.6%)
2	1544 (73.0%)
3	232 (11.0%)
4	9 (0.4%)
Any comorbidities	638 (30.2%)
Complication due to the tumor*	529 (25.0%)
Albumin level (g/L); mean (range)	38.39 (18.20–57.10)
Pre-operative hemoglobin (g/L); mean (range)	116 (39–186)
Neoadjuvant chemotherapy	224 (10.6%)
Tumor location	
Upper third	215 (10.2%)
Middle third	504 (23.8%)
Lower third	1308 (61.9%)
Mixed	87 (4.1%)
Type of resection	
Subtotal gastrectomy	1516 (71.7%)
Total gastrectomy	598 (28.3%)
Operative procedure	
Laparoscopy or laparoscopy-assisted	530 (25.1%)
Open	1584 (74.9%)
TNM stage^†^	
II	564 (26.7%)
III	1550 (73.3%)
Intraoperative blood loss (mL); mean (range)	208 (50–2300)
Operation time (min); mean (range)	202 (70–584)
Perioperative blood transfusion	507 (24.0%)
Postoperative complication^‡^	
Infection	148 (7.0%)
Non-infection	72 (3.4%)
Postoperative hospital stay (days)	11.13 (5–144)
Adjuvant chemotherapy cycles; median (25th–75th)	4 (1–6)

*Including pyloric obstruction or bleeding

^†^Tumor stages are based on 8th edition of the AJCC TNM classification

^‡^Defined as Clavien-Dindo grade II or greater. Patients who developed both infectious and noninfectious complications were classified into the infectious group

### Postoperative Infectious Complications

There were 220 patients (10.4%) who experienced a total of 291 adverse events within 30 days following surgery, including 170 (58.4%) cases of infection and 121 (41.6%) cases of noninfectious complications, defined as Clavien–Dindo grade II or greater (Supplementary Table 1). A total of 148 patients experienced 170 infections, with 92 cases of intra-abdominal infections (including those caused by leakage) being the most frequent, following by pulmonary (*n* = 61) and wound (*n* = 12) infections.

Age ≥ 65 years, BMI ≥ 25 kg/m^2^, positive smoking history, American Society of Anesthesiologist (ASA) score ≥ 3, comorbidity, preoperative hemoglobin < 100 g/L, preoperative albumin < 35 g/L, with complications due to the tumor, open procedure, longer operative time (≥ 240 min), larger intraoperative blood loss (≥ 300 mL), and BTF was all found as potential risk factors for postoperative infections by univariate analyses (all *P* values < 0.05; Table 2). After enrolling these factors into multivariate regression analysis, the final independent predictors for infections were found to be an operation time ≥ 240 min, perioperative BTF, open surgery, BMI ≥ 25 kg/m^2^, any comorbidity and a smoking history (Table 3). It is worthy to notice that NAC did not significantly increase the risk of postoperative infections (4.0% vs. 7.4%, *P* = 0.064) or total complications (7.6% vs. 10.7%, *P* = 0.150).

**Table 2:** Univariate analysis of possible predictors for post-operative infections following radical gastrectomy for stage II/III gastric cancer (*n* = 2114)

Variables	Infections (*n* = 148)	Noninfection (*n* = 1966)	*χ*^2^ value	*P* value
Gender			2.701	0.100
Male	108 (73.0%)	1305 (66.4%)		
Female	40 (27.0%)	661 (33.6%)		
Age (yr)			5.085	0.024
≥ 65	45 (30.4%)	439 (22.3%)		
< 65	103 (69.6%)	1527 (77.7%)		
Body mass index (kg/m^2^)			5.772	0.016
≥ 25	30 (20.3%)	260 (13.2%)		
< 25	118 (79.7%)	1706 (86.8%)		
Smoking history			5.518	0.019
Yes	78 (52.7%)	841 (42.8%)		
No	70 (47.3%)	1125 (57.2%)		
ASA score			7.378	0.007
≥ 3	27 (18.2%)	214 (10.9%)		
< 3	121 (81.8%)	1752 (89.1%)		
Comorbidity			7.084	0.008
Yes	59 (39.9%)	579 (29.5%)		
No	89 (60.1%)	1387 (70.5%)		
Preoperative hemoglobin (g/L)			4.922	0.027
≥ 100	100 (67.6%)	1489 (75.7%)		
< 100	48 (32.4%)	477 (24.3%)		
Preoperative albumin (g/L)			7.564	0.006
≥ 35	102 (68.9%)	1546 (78.6%)		
< 35	46 (31.1%)	420(21.4%)		
Complication due to the tumor*			5.544	0.019
Yes	49 (33.1%)	480(24.4%)		
No	99 (66.9%)	1486 (75.6%)		
Neo-adjuvant chemotherapy			3.424	0.064
Yes	9 (6.1%)	215 (10.9%)		
No	139 (93.9%)	1751 (89.1%)		
Operation method			7.695	0.006
Laparoscopy	23 (15.5%)	507 (25.8%)		
Open	125 (84.5%)	1459 (74.2%)		
Extent of gastric resection			0.370	0.124
Subtotal	98 (66.2%)	1418 (72.1%)		
Total	50 (33.8%)	548 (27.9%)		
Operation time (min)			49.521	< 0.001
≥ 240	68 (45.9%)	410 (20.9%)		
< 240	80 (54.1%)	1556 (79.1%)		
Intra-operative blood loss (mL)			8.607	0.003
≥ 300	46 (31.1%)	409 (20.8%)		
< 300	102 (68.9%)	1557 (79.2%)		
TNM stage			0.229	0.623
III	111 (75.0%)	1439 (73.2%)		
II	37 (25.0%)	527 (26.8%)		
Peri-operative blood transfusion			44.737	< 0.001
Yes	69 (46.6%)	438 (22.3%)		
No	79 (53.4%)	1528 (77.7%)		

*ASA* American Society of Anesthesiologist

*Including pyloric obstruction or bleeding

**Table 3:** Multivariate analysis of possible predictors for post-operative infections following radical gastrectomy for stage II/III gastric cancer (*n* = 2114)

Variables	Odds ratio [OR]	95% Confidence interval [CI]	*P* value
Operation time ≥ 240 min	2.981	2.095–4.240	< 0.001
Perioperative blood transfusion	2.739	1.927–3.895	< 0.001
Open surgery	1.939	1.215–3.093	0.005
Body mass index ≥ 25 kg/m^2^	1.757	1.130–2.733	0.012
Comorbidity	1.533	1.070–2.195	0.020
Smoking history	1.428	1.008–2.022	0.045

### Risk Factors for Poor CSS

Among the entire cohort of patients, 808 deaths (38.2%) occurred during the median follow-up period of 22 months (range 1–108), with a median overall survival (OS) time of 57 months. A total of 799 patients experienced tumor recurrences among whom 705 died due to GC (the exact causes of death of another 38 patients remain unknown).

In the univariate analysis, age ≥ 65 years, ASA score ≥ 3, TNM stage III, perioperative BTF, infections, and < 6 cycles of AC were found to be potential risk factors for poor CSS (all *P* values < 0.05; Table 4). Further multivariate Cox regression analysis confirmed that only stage III (hazard ratio (HR): 3.379, 95% confidence interval (CI) 2.712–4.211, *P* < 0.001), incomplete AC (HR 1.789, 95% CI 1.514–2.113, *P* < 0.001), infections (HR 1.323, 95% CI 1.013–1.727, *P* = 0.040), and BTF (HR 1.193, 95% CI 1.007–1.414, *P* = 0.042) were independent risk factors for a poorer CSS.

**Table 4:** Univariate and multivariate analyses of prognostic factors for cancer-specific survival (CSS) after radical gastrectomy of stage II/III gastric cancer (*n* = 2114)

Variables	*N*	Median CSS (months)	UV *P* value	MV HR (95% CI)	MV *P* value
Gender					
Male	1413 (66.8%)	69	0.908		
Female	701 (33.2%)	73			
Age (yr)					
≥ 65	484 (22.9%)	63	0.021		0.357
< 65	1630 (77.1%)	74			
ASA score					
≥ 3	241 (11.4%)	47	0.010		0.133
< 3	1873 (88.6%)	86			
Comorbidities					
Yes	638 (30.2%)	NA^†^	0.160		
No	1476 (69.8%)	65			
Hemoglobin (g/L)					
≥ 100	1589 (75.2%)	73	0.198		
< 100	525 (24.8%)	60			
Albumin (g/L)					
≥ 35	1648 (78.0%)	81	0.058		
< 35	466 (22.0%)	57			
TNM stage*					
III	1550 (73.3%)	44	< 0.001	3.379 (2.712–4.211)	< 0.001
II	564 (26.7%)	NA^†^			
Perioperative blood transfusion					
Yes	507 (24.0%)	48	0.001	1.193 (1.007–1.414)	0.042
No	1607 (76.0%)	81			
Postoperative infectious complication					
Yes	148 (7.0%)	33	0.006	1.323 (1.013–1.727)	0.040
No	1966 (93.0%)	79			
Adjuvant chemotherapy					
Incomplete	1401 (66.3%)	55	< 0.001	1.789 (1.514–2.113)	< 0.001
Complete	713 (33.7%)	NA^†^			

*ASA* American Society of Anesthesiologist; *CSS* cancer-specific survival; *CI* confidence interval; *HR* hazard ratio; *UV* univariate analysis; *MV* multivariate analysis

*Tumor stages are based on 8th edition of AJCC TNM classification

^†^Median cancer-specific survival time has not reached during the follow-up

### Association Between BTF, Infections, and CSS

**Fig. 1:**
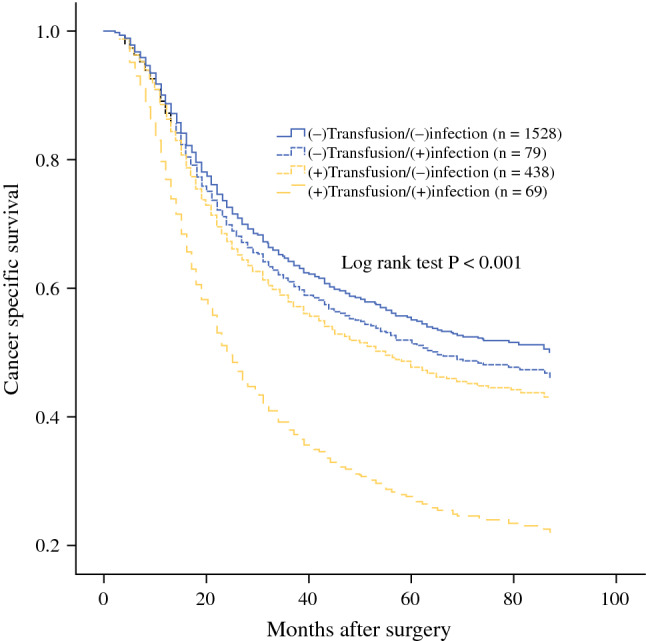
Cancer-specific survival curves in 2114 patients who underwent radical gastrectomy for stage II/III gastric cancer classified by receiving perioperative blood transfusion and experiencing postoperative infectious complications. (+) defined as receiving perioperative blood transfusion or experiencing postoperative infectious complications

Compared with the (−)transfusion/(−)infection (*n* = 1528), although the (−)transfusion/(+)infection group had comparable outcomes (*n* = 79, *P* = 0.576), the (+)transfusion/(−)infection group had poorer survival (*n* = 438, *P* = 0.024) and the (+)transfusion/(+)infection group was confirmed to have the worst outcomes (*n* = 69, *P* < 0.001) among the four groups (Fig. 1; Table 5). Additionally, a synergistic influence was confirmed in the (+)transfusion/(+)infection group compared to the (+)transfusion/(−)infection group (HR 1.767, 95% CI 1.206–2.590, *P* = 0.003). The same impact also was found with respect to OS and disease-free survival (DFS) (Supplementary Fig. 1).

**Table 5:** Multivariate analysis of cancer specific survival (CSS) of stage II/III gastric cancer following radical gastrectomy of the entire cohort (*n* = 2114)

Subgroups	*n* (%)	Median CSS (mo)	Hazard ratio (HR)	95% Confidence interval [CI]	*P* value
Blood transfusion/infection (−)Transfusion/(−)infection	1528 (72.3%)	81	Reference	Reference	
(−)Transfusion/(+)infection	79 (3.7%)	65	1.115	0.762–1.631	0.576
(+)Transfusion/(−)infection	438 (20.7%)	57	1.232	1.027–1.478	0.024
(+)Transfusion/(+)infection	69 (3.3%)	24	2.184	1.526–3.123	< 0.001

(+) Defined as receiving perioperative blood transfusion or developing postoperative infectious complications

### Benefit from Perioperative Chemotherapy

First, the potential predictors for incomplete AC were analyzed using univariate and multivariate logistic regression analysis. Age ≥ 65 years, ASA score ≥ 3, albumin < 35 g/L, and open surgery was identified to adversely affect the completion of AC (all *P* values < 0.05; Supplementary Table 2). In contrast, NAC was identified as an independent protective factor (HR 0.245, 95% CI 0.182–0.331, *P* < 0.001). Postoperative complications (28.6% vs. 34.3%, *P* = 0.092), severe complications (stage III-V assessed by Clavien–Dindo classification system) (29.3% vs. 33.9%, *P* = 0.412), infection (29.1% vs. 34.1%, *P* = 0.212), and intra-abdominal infections (26.1% vs. 34.1%, *P* = 0.113) all did not significantly have an impact on the completion of AC.

**Fig. 2:**
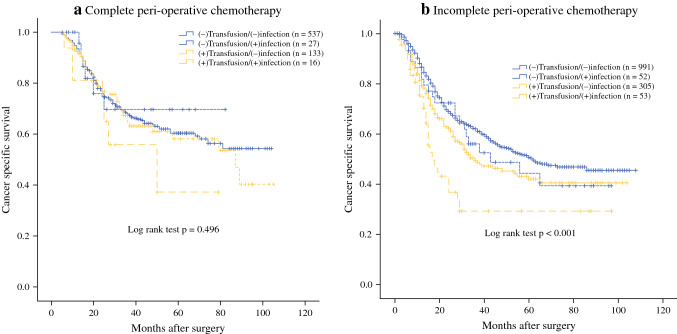
Cancer-specific survival curves in 2114 patients who underwent radical gastrectomy for stage II/III gastric cancer classified by receiving perioperative blood transfusion and experiencing post-operative infectious complications. **a**) Patients received complete perioperative chemotherapy (≥ 6 cycles, *n* = 713); **b**) Patients received incomplete perioperative chemotherapy (< 6 cycles, *n* = 1401). (+) defined as receiving perioperative blood transfusion or experiencing postoperative infectious complications

The median CSS for patients with complete AC (≥ 6 cycles, *n* = 713) was not reached during the follow-up, which was significantly longer than that in the incomplete AC group (< 6 cycles, *n* = 1401) (55 months, *P* < 0.001). Further stratified analysis revealed that regardless of experiencing BTF and infections or not, complete AC significantly improved CSS in stage II/III GC patients. In addition, CSS was comparable among patients with complete AC, whether they received a BTF and/or contracted an infection or not (*P* = 0.496, Fig. 2a). CSS was still different among the four groups for patients with incomplete AC (*P* < 0.001; Fig. 2b), just as for the entire cohort. Furthermore, in patients receiving NAC, CSS was similar among the four subgroups (*P* = 0.654) but differed significantly in those who did not undergo NAC (*P* < 0.001; Supplementary Fig. 2).

## Discussion

In this large cohort study of stage II/III GC patients from a single high-volume institution, we found that 24.0% of patients received perioperative BTF and 7.0% developed postoperative infections, which ranked as the most common complication after radical gastrectomy. The results are in keeping with our previous study and other researches from eastern countries.^6^^,^^22^^,^^23^ The incidence of postoperative complications, including infections, were reported to be significantly higher in studies from western countries.^24^ Possible reasons include patients in western countries usually had higher BMI values and had a longer duration of surgery, which are well-established predictors for postoperative infections.^6^ The retrospective nature and that only ≥ grade II complications were analyzed in the present study also might have decreased the incidence of infections reported. In addition, BTF was confirmed as an independent predictor for postoperative infection, as found in previous similar studies.^5^^,^^6^^,^^21^ One of our previous studies analyzed 2064 patients who underwent resection for GC. After adjusting for confounding biases, such as age and tumor stage using propensity score matching analysis, BTF was still confirmed to be significantly associated with infectious complications. Patients who were transfused with a high-volume (> 7.5 U) intraoperatively and those with leukocyte containing erythrocytes were found to be at the highest risk.^6^

Although perioperative chemotherapy (chemoradiation combined therapy in some western countries) has been recommended as the standard treatment for locally advanced GC, only 713 (33.7%) of 2114 patients received at least 6 courses of AC. Some scholars have investigated the risk factors for noncompletion of AC and older age (> 65 years), body weight loss, creatinine clearance < 60 mL/min, poor immunological and nutritional status were identified as independent predictors.^19^^,^^25^^–^27 In a retrospective analysis containing 765 GC patients from U.S. Gastric Cancer Collaborative, Squires et al. found that transfused patients were less likely to receive AC, but whether transfusion was an independent predictor for poor compliance with AC has not described.^9^ In the present study, although patients receiving BTF seemed unlikely to complete 6 cycles of AC (29.4% vs. 35.8%, *P* = 0.018, Supplementary Table 2), it lost its significance in further multivariate analyses (*P* = 0.858), which was consistent with our previous study.^19^ Thus, it seemed that BTF-caused poor prognosis of GC patients was not due to effects on noncompletion of AC.

With respect to survival, both BTF and infections were confirmed to have an adverse impact on CSS. More importantly, and for the first time, a synergistic adverse influence of both receiving BTF and developing infections on the CSS of GC patients was confirmed, which was echoed by the results of Aquina and colleagues.^21^ In their study of 24,230 stage I to III colon cancer cases, after risk adjustment, BTF and sepsis were found to be associated with poorer OS, CSS, and cardiovascular disease-specific survival. Additionally, there was an additive effect in those patients who experienced both BTF and sepsis. This finding can be partly explained by systemic inflammation and depression of host immune response during an operation, transfusion, and infections that accelerate cancer cell proliferation and invasion.^21^^,^^28^^,^^29^ Because BTF has an independent, and also a synergistic influence with regard to infection on prognosis after curative resection for GC, to reduce the risk of infection and improve survival, restrictive transfusion practice seems to be a promise strategy. In fact, some prospective, multicenter, large-scale studies have compared short and long outcomes after restrictive (hemoglobin < 70 or 75 g/L) or liberal (hemoglobin < 90 or 85 g/L) levels during surgery or after hematopoietic cell transplantation and concluded that a restrictive transfusion strategy would reduce the number of transfusions required without impacting on the mortality rate.^30^^,^^31^ It seems inappropriate to copy their experiences verbatim in patients who underwent gastrectomy and, more importantly, whether different BTF strategies would influence the prognosis of patients with malignancy, but this issue has rarely been investigated. Preoperative iron supplementation and transfusion with leukocyte-depleted red blood cells also may be useful strategies to reduce the requirement for transfusion and the rate of occurrence of infections. To establish whether these measures improve survival, further prospective studies are needed.^6^^,^^32^

Postoperative complications have been identified to have an adverse impact on completion of AC, but the causative mechanism remains unclear. Whether complete AC can mitigate the adverse influence of complications on prognosis has rarely been investigated. In a recent study that analyzed 206 patients who underwent curative resection for locally advanced GC, it was found that the 3-year OS of patients without postoperative complication was 62.1%, which was significantly better than 56.9% and 33.3% in patients experienced a minor (stage I-II assessed by Clavien-Dindo classification system) or a major (stage III-IV) complication, respectively. Further analysis revealed that patients who suffered a major complication were less likely to complete multimodality therapy.^12^ In particular, infectious complications has confirmed as a significant predictor for noncompletion of 1-year adjuvant S-1 monotherapy for GC in a multi-institution study.^27^ In the present study, however, the difference of completion of AC was slightly outside the significance level between patients who experienced postoperative complications or did not (*P* = 0.092). Additionally, severe complications, infection, and intra-abdominal infections were all identified to have insignificant influence on compliance with of AC. These results differed from previous studies, but the relatively low incidence of postoperative complications and low completion of AC may have had an influence on the statistical power of our model. Additionally, the economic burden also might act as a confounding factor when investigating the potential risk factors for incomplete AC in China, as a developing country. Thus, a further prospective study with a larger cohort of patients is needed.

In the present study, the median CSS of patients who experienced both BTF and infection improved from 17 to 50 months if they completed at least 6 cycles of AC and became comparable to patients who had either a BTF or an infection or neither (*P* = 0.496; Fig. 2a). The median CSS of patients who had both a BTF and infection was still significantly shorter if the planned AC was incomplete (*P* < 0.001; Fig. 2b). These findings strongly support the completion of AC, particularly for patients who receive a BTF and/or develop infections. In concordance with the results of our study, Li et al. reported that completion of multimodality therapy could extenuate the adverse influence of complications on the long-term survival of locally advanced GC patients.^12^ Another retrospective study conducted by Vicente and colleagues confirmed that preoperative therapy for GC was protective for poor oncological outcomes in patients with complications after gastrectomy.^33^ It was echoed by Hayashi et al., who argued that NAC could cancel out the negative influence of infections on prognosis in advanced GC patients.^34^ Elimination of micrometastases by NAC before postoperative complications occur may suppress the growth of residual invisible cancer cells and control metastasis. Thus, in order to complete AC and negate the impact of complications on survival, there may be some room for consideration of performing NAC (instead of postoperative chemotherapy), especially in patients at high risk of suffering from postoperative complications.^12^^,^^33^^,^^34^ Further prospective studies are still necessary to clarify this supposition.

Only 224 patients (10.6%) received NAC in the present study, which was significantly lower than that reported in Western countries.^33^ The possible explanation was D2 gastrectomy followed by AC was considered to be a standard in the East (China, Japan, etc.), whereas NAC (or neoadjuvant radiochemotherapy) is recommended as standard treatment in the West.^35^ Briefly speaking, large type 3 or 4 tumors, and/or bulky nodal disease, was the main target of NAC in our institution. As shown in Supplementary Fig. 2B, the prognosis were comparable among those receiving BTF or not, developing infection or not. The negative results may be due to the extremely small sample size (*n* = 4 and 5 in (−)transfusion/(+)infection and (+)transfusion/(+)infection group, respectively), thus dramatically increases the possibility of a type II error and limits the statistical power. Thus, further prospective studies with a large cohort of patients are still needed to clarify the interaction among BTF, infection, and prognosis in patients undergoing NAC.

Although some important findings have been reported, the present study has a number of limitations. First and principally, it was a retrospective and single institution study. Thus, it is possible that some complications (particularly stage I-II) might have been missed and selection bias was inevitable. Second, a growing body of evidence shows that NAC can improve the R0 resection rate, completion of AC, and prognosis in patients with locally advanced GC. Only 224 patients (10.6%) received NAC in our study, which may harm the generalizability of our findings, especially in Western countries, where NAC (or radiochemotherapy) is recommended as standard treatment. Third, several anticancer regimens were used in our institution during the study, such as oxaliplatin plus S-1, oxaliplatin plus capecitabine, or S-1 alone. This also may have an impact on the completion of AC and prognosis, as a result, becoming a confounding factor. Fourth, the median follow-up period (22 months) was relatively short, but more than 90% of recurrences were diagnosed within 2 years of surgery.^36^ Notwithstanding the above-mentioned limitations, the present study nevertheless first investigated the interaction of BTF, infections and AC on survival of stage II/III GC patients after radical gastrectomy, based on a relatively large cohort of patients.

## Conclusions

The present study from a high-volume center in China has revealed that BTF was a significant predictor for postoperative infectious complications following radical gastrectomy for stage II/III GC. Both BTF and infections had an adverse impact on CSS and a synergistic influence was confirmed in patients who had a BTF or experienced infections. More importantly, we found that completion of AC could compensate for the adverse influence of BTF and infections on prognosis. These findings strongly support the need for patients with locally advanced GC to complete AC, particularly patients who have received a BTF and/or developed infections. NAC seemed to be a feasible strategy for treatment, but further prospective studies are required.

## Electronic supplementary material

Below is the link to the electronic supplementary material.

Supplementary material 1 (DOC 33 kb)

Supplementary material 2 (DOC 73 kb)

**Supplementary Fig.** **1** Survival curves in 2114 patients who underwent radical gastrectomy for stage II/III gastric cancer classified by receiving perioperative blood transfusion and experiencing postoperative infectious complications. **A)** Disease-free survival; **B)** Overall survival. (+) defined as receiving perioperative blood transfusion or experiencing postoperative infectious complications (TIFF 5534 kb)

**Supplementary Fig.** **2** Survival curves in 2114 patients who underwent radical gastrectomy for stage II/III gastric cancer classified by receiving neoadjuvant chemotherapy or not. **A)** Patients received no neoadjuvant chemotherapy (*n* = 1890); **B)** Patients received neoadjuvant chemotherapy (*n* = 224) (TIFF 5344 kb)
