# Supplementary material for: Association Among Blood Transfusion, Postoperative Infectious Complications, and Cancer-Specific Survival in Patients with Stage II/III Gastric Cancer After Radical Gastrectomy: Emphasizing Benefit from Adjuvant Chemotherapy
Source: Ann Surg Oncol. 2020 Sep 14;28(4):2394–404. doi: 10.1245/s10434-020-09102-4 (PMC7940152; doi:10.1245/s10434-020-09102-4)
Supplement: Supplementary file 1 — Supplementary material 1 (DOC 33 kb) [file 10434_2020_9102_MOESM1_ESM.doc]

| **Supplementary Table 1.** Post-operative complications of the entire 2,114 patients ( n = 291) | |
| --- | --- |
| Complications | Number(%) |
| Infectious | 170 (58.4%) |
| Intra-abdominal infection | 92 (31.6%) |
| Pneumonia | 61 (21.0%) |
| Wound infection | 12 (4.1%) |
| Sepsis | 3 (1.0%) |
| Urinary tract infection | 2 (0.7%) |
| Non-infectious | 121 (41.6%） |
| Pleural effusion | 28 (9.6%) |
| Ascites | 24 (8.2%) |
| Intestinal obstruction | 19 (6.5%) |
| Gastrointestinal bleeding | 19 (6.5%) |
| Intra-abdominal bleeding | 18 (6.2%) |
| Cerebral infarction | 4 (1.4%) |
| Anastomotic stricture | 3 (1.0%) |
| Pneumothorax | 3 (1.0%) |
| Delayed gastric emptying | 2 (0.7%) |
| Liver failure | 2 (0.7%) |
| Cardiac arrest | 2 (0.7%) |
| Urinary retention | 2 (0.7%) |
| Renal failure | 2 (0.7%) |
| Diabetic ketoacidosis | 1 (0.3%) |
| Acute attack of chronic obstructive pulmonary disease | 1 (0.3%) |
| Iatrogenic common bile duct injury | 1 (0.3%) |
