# Supplementary material for: Association Among Blood Transfusion, Postoperative Infectious Complications, and Cancer-Specific Survival in Patients with Stage II/III Gastric Cancer After Radical Gastrectomy: Emphasizing Benefit from Adjuvant Chemotherapy
Source: Ann Surg Oncol. 2020 Sep 14;28(4):2394–404. doi: 10.1245/s10434-020-09102-4 (PMC7940152; doi:10.1245/s10434-020-09102-4)
Supplement: Supplementary file 2 — Supplementary material 2 (DOC 73 kb) [file 10434_2020_9102_MOESM2_ESM.doc]

| **Supplementary Table 2a.** Association between clinicopathological characteristics and completeness of peri-operative chemotherapy for stage II/III gastric cancer (n = 2,114) | | | |
| --- | --- | --- | --- |
| Variables | Complete AC  (n = 713 ) | Incomplete AC  (n = 1401 ) | *P* value |
| Gender |  |  | 0.219 |
| Male | 464(65.1%) | 949(67.7%) |  |
| Female | 249(34.9%) | 452(32.3%) |  |
| Age (years) |  |  | < 0.001 |
| ≥ 65 | 87(12.2%) | 397(28.3%) |  |
| < 65 | 626(87.8%) | 1004(71.7%) |  |
| Body mass index (kg/m^2^) |  |  | 0.112 |
| ≥ 18.5 | 629(88.2%) | 1201(85.7%) |  |
| < 18.5 | 84(11.8%) | 200(14.3%) |  |
| Hemoglobin (g/L) |  |  | 0.452 |
| ≥ 100 | 543(76.2%) | 1046(74.7%) |  |
| < 100 | 170(23.8%) | 355(25.3%) |  |
| ASA score |  |  | < 0.001 |
| ≥ 3 | 37(5.2%) | 204(14.6%) |  |
| < 3 | 676(94.8%) | 1197(85.4%) |  |
| Comorbidity |  |  | 0.222 |
| Yes | 203(28.5%) | 435(31.0%) |  |
| No | 510(71.5%) | 966(69.0%) |  |
| Albumin level (g/L) |  |  | < 0.001 |
| ≥ 35 | 601(84.3%) | 1047(74.7%) |  |
| < 35 | 112(15.7%) | 354(25.3%) |  |
| Neo-adjuvant chemotherapy |  |  | < 0.001 |
| Yes | 146(20.5%) | 78(5.6%) |  |
| No | 567(79.5%) | 1323(94.4%) |  |
| Complication due to the tumor |  |  | 0.039 |
| Yes | 159(22.3%) | 370(26.4%) |  |
| No | 554(77.7%) | 1031(73.6%) |  |
| Operation method |  |  | < 0.001 |
| Open | 481(67.5%) | 1103(78.7%) |  |
| Laparoscopy | 232(32.5%) | 298(21.3%) |  |
| Extent of gastric resection |  |  | 0.975 |
| Subtotal | 511(71.7%) | 1005(71.7%) |  |
| Total | 202(28.3%) | 396(28.3%) |  |
| Operation time (min) |  |  | 0.065 |
| ≥ 240 | 178(25.0%) | 300(21.4%) |  |
| < 240 | 535(75.0%) | 1101(78.6%) |  |
| Intra-operative blood loss (mL) |  |  | 0.470 |
| ≥ 300 | 147(20.6%) | 308(22.0%) |  |
| < 300 | 566(79.4%) | 1093(78.0%) |  |
| pTNM stage ^†^ |  |  | < 0.001 |
| III | 557(78.1%) | 993(70.9%) |  |
| II | 156(21.9%) | 408(29.1%) |  |
| Peri-operative blood transfusion |  |  | 0.018 |
| Yes | 149(20.9%) | 358(25.6%) |  |
| No | 564(79.1%) | 1043(74.4%) |  |
| Post-operative complications ^‡^ |  |  | 0.092 |
| Yes | 63(8.8%) | 157(11.2%) |  |
| No | 650(91.2%) | 1244(88.8%) |  |
| ASA, American Society of Anesthesiologist.  * Including pyloric obstruction or bleeding.  † Tumor stages are based on 8th edition of the AJCC TNM classification.  ‡ Defined as Clavien-Dindo grade II or greater. | | | |
|  | | | |
| **Supplementary Table 2b.** Multivariate analysis of possible predictors for completeness of peri-operative chemotherapy for stage II/III gastric cancer (n = 2,114) | | | |
| Variables | Odds Ratio [OR] | 95% CI | *P* value |
| Age ≥ 65 years | 2.381 | 1.821-3.112 | < 0.001 |
| American Society of Anesthesiologist score ≥ 3 | 1.819 | 1.238-2.673 | 0.002 |
| Albumin < 35g/L | 1.430 | 1.114-1.836 | 0.005 |
| Neo-adjuvant chemotherapy | 0.245 | 0.182-0.331 | < 0.001 |
| Open surgery | 1.641 | 1.324-2.032 | < 0.001 |
| CI, Confidence Interval. | | | |
