# Supplementary figures and images for: Association Among Blood Transfusion, Postoperative Infectious Complications, and Cancer-Specific Survival in Patients with Stage II/III Gastric Cancer After Radical Gastrectomy: Emphasizing Benefit from Adjuvant Chemotherapy
Source: Ann Surg Oncol. 2020 Sep 14;28(4):2394–404. doi: 10.1245/s10434-020-09102-4 (PMC7940152; doi:10.1245/s10434-020-09102-4)

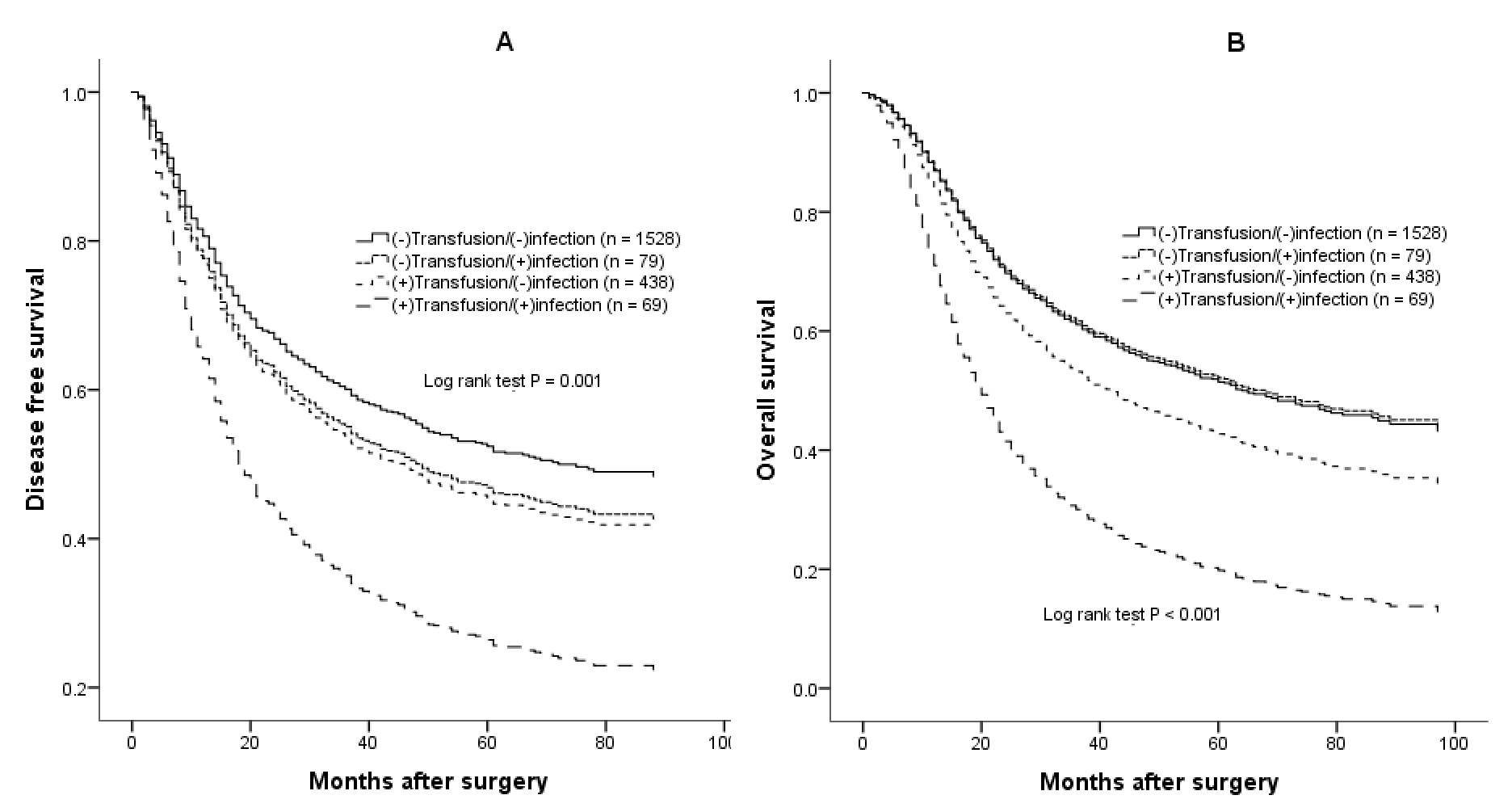

Supplement: Supplementary file 3 — Supplementary Fig. 1 Survival curves in 2114 patients who underwent radical gastrectomy for stage II/III gastric cancer classified by receiving perioperative blood transfusion and experiencing postoperative infectious complications. A) Disease-free survival; B) Overall survival. (+) defined as receiving perioperative blood transfusion or experiencing postoperative infectious complications (TIFF 5534 kb) [file 10434_2020_9102_MOESM3_ESM.tif]

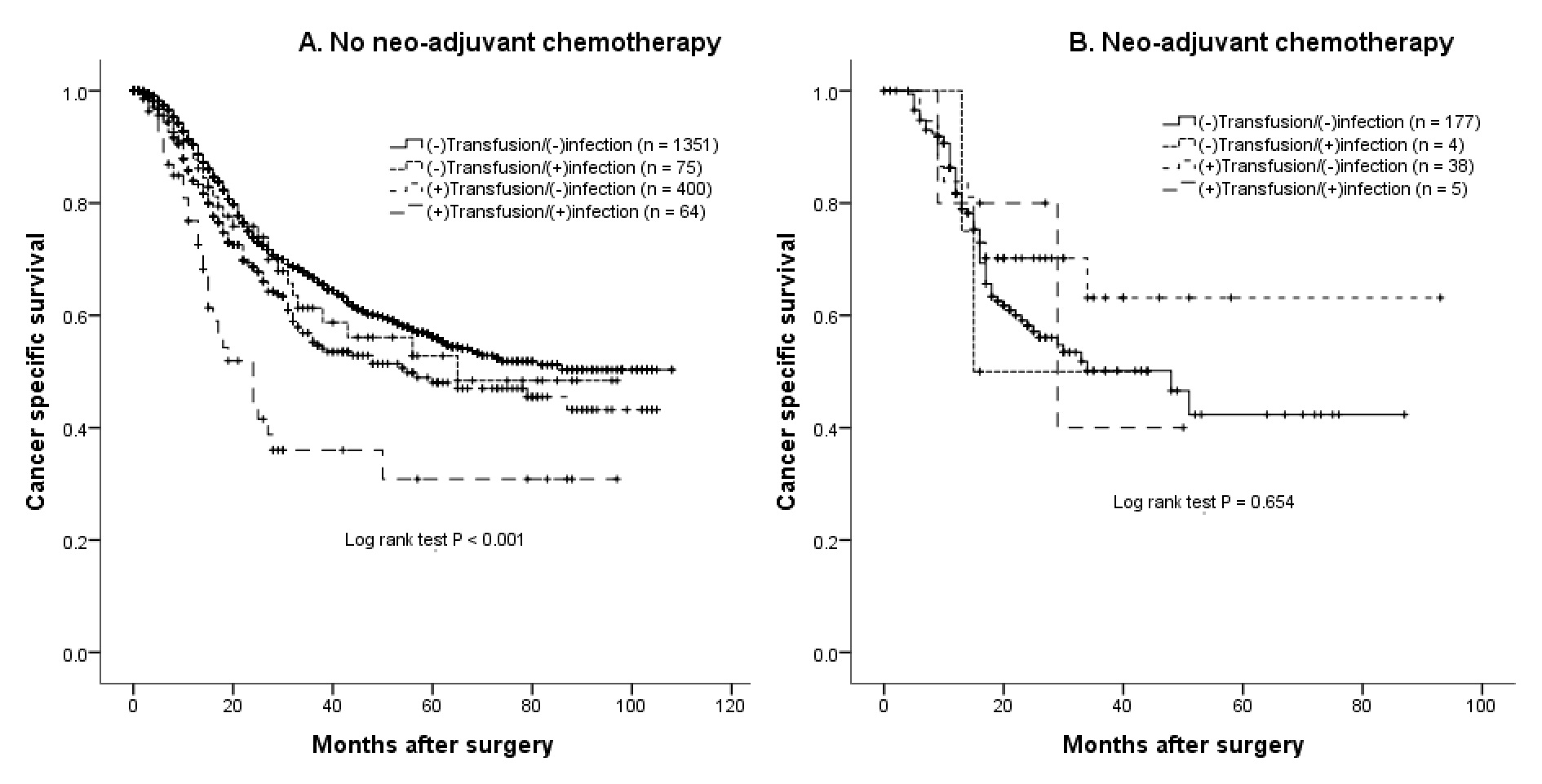

Supplement: Supplementary file 4 — Supplementary Fig. 2 Survival curves in 2114 patients who underwent radical gastrectomy for stage II/III gastric cancer classified by receiving neoadjuvant chemotherapy or not. A) Patients received no neoadjuvant chemotherapy (n = 1890); B) Patients received neoadjuvant chemotherapy (n = 224) (TIFF 5344 kb) [file 10434_2020_9102_MOESM4_ESM.tif]
